# Breast cancer risk assessment with five independent genetic variants and two risk factors in Chinese women

**DOI:** 10.1186/bcr3101

**Published:** 2012-01-23

**Authors:** Juncheng Dai, Zhibin Hu, Yue Jiang, Hao Shen, Jing Dong, Hongxia Ma, Hongbing Shen

**Affiliations:** 1 National Key Laboratory of Reproductive Medicine, Nanjing Medical University, Nanjing 210029, China; 2 Department of Epidemiology and Biostatistics and Ministry of Education Key Lab for Modern Toxicology, School of Public Health, Nanjing Medical University, Nanjing 210029, China; 3 Section of Clinical Epidemiology, Jiangsu Key Lab of Cancer Biomarkers, Prevention and Treatment, Cancer Center, Nanjing Medical University, Nanjing 210029, China

## Abstract

**Introduction:**

Recently, several genome-wide association studies (GWAS) have identified novel single nucleotide polymorphisms (SNPs) associated with breast cancer risk. However, most of the studies were conducted among Caucasians and only one from Chinese.

**Methods:**

In the current study, we first tested whether 15 SNPs identified by previous GWAS were also breast cancer marker SNPs in this Chinese population. Then, we grouped the marker SNPs, and modeled them with clinical risk factors, to see the usage of these factors in breast cancer risk assessment. Two methods (risk factors counting and odds ratio (OR) weighted risk scoring) were used to evaluate the cumulative effects of the five significant SNPs and two clinical risk factors (age at menarche and age at first live birth).

**Results:**

Five SNPs located at 2q35, 3p24, 6q22, 6q25 and 10q26 were consistently associated with breast cancer risk in both testing set (878 cases and 900 controls) and validation set (914 cases and 967 controls) samples. Overall, all of the five SNPs contributed to breast cancer susceptibility in a dominant genetic model (2q35, rs13387042: adjusted OR = 1.26, *P *= 0.006; 3q24.1, rs2307032: adjusted OR = 1.24, *P *= 0.005; 6q22.33, rs2180341: adjusted OR = 1.22, *P *= 0.006; 6q25.1, rs2046210: adjusted OR = 1.51, *P *= 2.40 × 10^-8^; 10q26.13, rs2981582: adjusted OR = 1.31, *P *= 1.96 × 10^-4^). Risk score analyses (area under the curve (AUC): 0.649, 95% confidence interval (CI): 0.631 to 0.667; sensitivity = 62.60%, specificity = 57.05%) presented better discrimination than that by risk factors counting (AUC: 0.637, 95% CI: 0.619 to 0.655; sensitivity = 62.16%, specificity = 60.03%) (*P *< 0.0001). Absolute risk was then calculated by the modified Gail model and an AUC of 0.658 (95% CI = 0.640 to 0.676) (sensitivity = 61.98%, specificity = 60.26%) was obtained for the combination of five marker SNPs, age at menarche and age at first live birth.

**Conclusions:**

This study shows that five GWAS identified variants were also consistently validated in this Chinese population and combining these genetic variants with other risk factors can improve the risk predictive ability of breast cancer. However, more breast cancer associated risk variants should be incorporated to optimize the risk assessment.

## Introduction

Breast cancer is one of the most common cancers among women worldwide [1]. Although life/environment related factors are implicated in breast carcinogenesis, it is a complex polygenic disorder in which genetic makeup also plays an important role [2,3]. In the past decades, high-penetrance genes (for example, *BRCA1, BRCA2, PTEN *and *TP53*) have been identified to be associated with familiar breast cancer [4]. However, these genes account for less than 5% of overall breast cancer patients and most of the risk is likely to be attributable to more low-penetrance genetic variants [5-7].

Recently, several genome-wide association studies (GWAS) reported many novel breast cancer predisposing single nucleotide polymorphisms (SNPs) [8-14]. However, most of the studies were conducted among Caucasians [8-13] and only one among Chinese [14], and whether these genetic variants are applicable marker SNPs in Asian women is unclear. Furthermore, evaluation of a risk-predicting model is an important topic in genetic studies of human diseases, including breast cancer. An effective risk-predicting model can assist physicians in disease prevention, diagnosis, prognosis and treatment [15]. For the harvest of GWAS on breast cancer, many studies combined the genetic markers and other traditional risk factors together to evaluate the risk-predicting model of breast cancer [16-22]. However, most of the breast cancer risk model effects are unsatisfied and only one related study was available in Chinese women [17].

In the current study, a two-stage case-control study of 1,792 breast cancer cases and 1,867 cancer-free controls was conducted among Chinese women to replicate 15 selected SNPs identified from previous GWAS. Then, risk models were constructed and absolute risk was calculated to evaluate the combined effects of the significant SNPs and clinical risk factors.

## Materials and methods

### Study subjects

This study was approved by the institutional review board of Nanjing Medical University. The hospital-based case-control study included 1,792 breast cancer cases and 1,867 cancer-free controls, and the detail process of subjects recruitment was described previously [23-25]. In brief, incident breast cancer patients were consecutively recruited from the First Affiliated Hospital of Nanjing Medical University, the Cancer Hospital of Jiangsu Province and the Gulou Hospital, Nanjing, China, between January 2004 and April 2010. Exclusion criteria included reported previous cancer history, metastasized cancer from other organs, and previous radiotherapy or chemotherapy. All breast cancer cases were newly-diagnosed and histopathologically confirmed, without restrictions of age or histological types. Cancer-free control women, frequency-matched to the cases on age (± 5 years) and residential area (urban or rural), were randomly selected from a cohort of more than 30,000 participants in a community-based screening program for non-infectious diseases conducted in the same region. All participants were ethnic Han Chinese women. Of the eligible participants, 878 cases and 900 controls were randomly assigned to form the testing set, and the remaining 914 cases and 967 controls formed the validation set.

After providing informed consent, each woman was personally interviewed face-to-face by trained interviewers using a pre-tested questionnaire to obtain information on demographic data, menstrual and reproductive history, and environmental exposure history. After the interview, each subject provided 5 ml of venous blood. The estrogen receptor (ER) and progesterone receptor (PR) status of breast cancer was determined by immunohistochemistry examinations which were obtained from the medical records of the hospitals.

### SNP selection and genotyping

The SNP selection procedure followed three criteria: (a) reported marker SNP in previous GWAS (last search in November 2009); (b) minor allele frequency (MAF) ≥ 0.05 in Chinese Han Beijing (CHB) based on the HapMap database (phase II, released 24 in November 2008); (c) only SNPs with low linkage disequilibrium (LD) were included (r^2 ^< 0.8) if multiple SNPs can be found at the same region. Overall, 15 SNPs (11 regions of 2q35, 3p24, 5p11, 5p12, 6q22, 6q25, 8q24, 10q26, 11p15, 16q12 and 17q23; Table 1) were selected and genotyped by using the middle-throughput TaqMan OpenArray Genotyping Platform (Applied Biosystems Inc., Carlsbad, CA, USA) for testing set samples (878 cases and 900 controls) and by TaqMan Assays on ABI PRISM 7900 HT Platform (Applied Biosystems Inc.) for validation set samples (914 cases and 967 controls). For OpenArray Assays, normalized human DNA samples were loaded and amplified on customized arrays following the manufacturer's instructions. Each 48-sample array chip contained two NTCs (no template controls). For TaqMan Assays, approximately equal numbers of case and control samples were assayed in each 384-well plate. Two blank controls in each plate were used for quality control and 96 duplicates were randomly selected to repeat for the two platforms, and the results were more than 97% concordant.

**Table 1:** Association of breast cancer risk with 15 SNPs selected from previous GWAS study in the Testing Set.

SNP	**Chr**. (Cytoband)	Position	Associated genes	GWAS study	Alleles^a^	Call Rate (%)	*P * ^b^	MAF^c^	MAF^d^	Case (%)	Control (%)	*P * ^e^
**rs13387042**	2q35	217614077	TNP1, IGFBP5, IGFBP2	Stacey, 2007 (12) Thomas, 2009 (13)	G > A	98.65	0.41	0.11	0.12	1.39/25.87/72.74	1.01/21.08/77.91	0.039
**rs4973768**	3p24.1	27391017	SLC4A7	Ahmed, 2009 (8)	C > T	97.58	0.91	0.17	0.19	3.05/35.05/61.90	3.63/31.52/64.85	0.265
**rs2307032**	3p24.1	27407999	SLC4A7	Ahmed, 2009 (8)	C > T	97.92	0.08	0.41	0.40	18.58/50.64/30.78	17.61/45.23/37.16	0.017
**rs16886165**	5p11.2	56058840	MAP3K1	Thomas, 2009 (13)	T > G	96.34	0.65	0.31	0.34	12.10/46.98/40.93	11.49/45.98/42.53	0.781
**rs889312**	5q11.2	56067641	MAP3K1	Easton, 2007 (9)	C > A	98.31	0.23	0.50	0.48	21.55/50.41/28.04	22.03/52.09/25.88	0.595
**rs4415084**	5p12	44698272	Unknown	Stacey, 2008 (12) Thomas,2009 (13)	A > G	96.12	0.03	0.46	0.43	19.95/44.56/35.84	20.41/45.64/33.94	0.798
**rs10941679**	5p12	44742255	MRPS30	Stacey, 2008 (12) Thomas,2009 (13)	G > A	97.19	0.95	0.57	0.50	24.23/49.29/26.48	24.83/50.23/24.94	0.768
**rs2180341**	6q22.33	127642323	ECHDC1, RNF146	Gold, 2008 (10)	A > G	97.30	0.49	0.22	0.26	6.35/43.88/49.76	7.50/37.95/54.55	0.040
**rs2046210**	6q25.1	151990059	ESR1, C6orf97	Zheng, 2009 (14)	G > A	98.48	0.50	0.35	0.34	18.35/47.97/33.68	12.36/44.16/43.48	1.26 × 10^-5^
**rs13281615**	8q24.21	128424800	Unknown	Easton, 2007 (9)	A > G	97.98	0.59	0.57	0.49	23.56/52.40/24.03	24.63/49.04/26.32	0.353
**rs1562430**	8q24.21	128457034	Unknown	Thomas 2009 (13)	T > C	98.82	0.11	0.20	0.18	2.75/27.38/69.87	2.49/31.33/66.18	0.191
**rs2981582**	10q26.13	123342307	FGFR2	Easton, 2007 (9)	C > T	99.44	0.94	0.33	0.31	12.61/45.87/41.51	9.82/43.3/46.88	0.037
**rs3817198**	11p15.5	1865582	LSP1	Easton, 2007 (9)	T > C	98.82	0.88	0.09	0.12	2.41/23.79/73.79	1.58/21.53/76.89	0.213
**rs12443621**	16q12.1	51105538	TNRC9	Easton, 2007 (9)	G > A	99.10	0.34	0.39	0.45	18.23/48.17/33.60	19.10/51.12/29.78	0.227
**rs6504950**	17q23.2	50411470	COX11	Ahmed, 2009 (8)	G > A	98.71	0.84	0.10	0.09	0.35/14.14/85.52	0.78/15.92/83.30	0.264

^a ^Major > Minor; ^b ^*P-*values for Hardy-Weinberg equilibrium(HWE) in the controls by exact test; ^c ^Minor Allele Frequency (MAF) of Chinese Han population from Beijing (CHB) based on the International HapMap project (Phase II+III, rel 27); ^d ^Minor Allele Frequency (MAF) in controls; ^e ^*P-*values for genotypic frequency between cases and controls by Fisher's exact test.

### Statistical analyses

Differences between breast cancer cases and controls in demographic characteristics, risk factors and frequencies of SNPs were evaluated by Fisher's exact tests (for categorical variables) or Student *t-*test or *t'*-test (equal variances not assumed) (for continuous variables). Hardy-Weinberg equilibrium was evaluated by exact test among the controls [26].

As shown in Additional file 1, three steps were performed to assess the breast cancer risk model. (1) SNPs screening. Following a two-stage strategy, associations between SNPs and risk of breast cancer were estimated by computing odds ratios (ORs) and their 95% confidence intervals (CIs). (2) Risk model construction. For the model parsimony, only genetic or clinical risk factors that were independently associated with breast cancer were included. Both OR (odds ratio) and AR (absolute risk) were taken as indicators to evaluate the risk model. For the OR-based risk model, two different methods were used. One method treated each risk allele/factor equally and combined them based on the counts of risk alleles/factors. Another method assessed the effects of the SNPs and risk factors using a risk score analysis with a linear combination of the SNP genotypes or risk factors weighted by their individual OR (The log odds at each SNP locus was additive in the number of minor alleles, and the log odds for the entire model was additive across SNPs and other risk factors). Then the risk score was classified into four groups by its quartiles in controls. AR is the risk of developing a disease over a time-period. In our paper, the AR for each woman was estimated by a modified Gail model [16,27]. This method is described as a multiplicative model used to derive genotype relative risk from the allelic OR. The allelic OR for each SNP was obtained assuming an additive genetic model by logistic regression analysis. For each of the three genotypes at each SNP, the genotype relative risk was converted to the risk relative to the population. The overall risk relative to the population was derived by combining the risks relative to the population of all SNPs as well as the two clinical risk factors (age at menarche and age at first live birth) of the individual by multiplication. Finally, the AR for each woman was obtained based on the overall risk relative to the population, calibrated by the incidence rate of breast cancer for women (aged 20 to 85 years), and the mortality rate for all causes except breast cancer from the Shanghai registration system, China [28]. (3) Risk model discrimination. The model performance was evaluated by receiver-operator characteristic (ROC) curves and the area under the curve (AUC) to classify the breast cancer cases and controls. The difference of AUCs was tested by a non-parametric approach developed by DeLong ER *et al. *[29]. Furthermore, for the absolute risk-based risk models, we used the 10-fold cross-validation method to check the reliability of the models. All of the statistical analyses were two-sided and performed with Statistical Analysis System software (9.1.3; SAS Institute, Cary, NC, USA) and Stata (9.2; StataCorp LP, Lakeway Drive College Station, TX, USA), unless indicated otherwise.

## Results

A total of 1,792 breast cancer cases and 1,867 cancer-free controls were included in the final analysis, and the characteristics of these subjects were summarized in Table 2. Age at menarche (*P *< 0.001) and age at first live birth (*P *< 0.001) were consistently, differentially distributed between the cases and the controls in all samples. Among 1,437 breast cancer cases with known ER and PR status, 662 (46.07%) were both ER and PR positive, and 498 (34.66%) were both negative.

**Table 2:** Distribution of demographic characteristics and known breast cancer risk factors for cases and controls included in the study

Variables	Testing Set, TS (*n *= 1,778)		Validation Set, VS (*n *= 1,881)		TS + VS (*n *= 3,659)		
	
	Cases (*n *= 878)	Controls (*n *= 900)	Cases (*n *= 914)	Controls (*n *= 967)	Cases (*n *= 1,792)	Controls (*n *= 1,867)	*P-*value
**Age, yr (Mean ± SD)**	51.29 ± 11.38	51.47 ± 11.67	50.11 ± 11.36	48.64 ± 12.28	50.69 ± 11.38	50.01 ± 12.07	0.08
**Age group, yr (%)**							0.06
**< 50**	425 (48.41)	463 (51.44)	467 (51.09)	526 (54.40)	896 (49.78)	989 (52.97)	
**≥ 50**	453 (51.59)	437 (48.56)	447 (48.91)	441 (45.60)	900 (50.22)	878 (47.03)	
**Age at menarche, yr (Mean ± SD)**	15.26 ± 1.83	15.85 ± 1.89	15.19 ± 1.94	16.23 ± 1.81	15.22 ± 1.89	16.05 ± 1.86	< 0.001
**Age at menarche group, yr (%)**							< 0.001
**< 15 (Early menarche)**	331 (38.40)	227 (25.33)	357 (39.98)	169 (17.57)	688 (39.20)	396 (21.31)	
**15 to 17 (Normal menarche)**	325 (37.70)	352 (39.29)	333 (37.29)	376 (39.09)	658 (37.49)	728 (39.18)	
**≥ 17 (Late menarche)**	206 (23.90)	317 (35.38)	203 (22.73)	417 (43.35)	409 (23.30)	734 (39.50)	
**Age at first live birth, yr (Mean ± SD)**	25.62 ± 3.39	24.90 ± 3.35	25.51 ± 3.06	24.17 ± 2.51	25.56 ± 3.22	24.52 ± 2.99	< 0.001^d^
**Age at first live birth group, yr (%)^a^**							< 0.001
**< 25 (Early birth)**	305 (34.74)	398 (44.27)	312 (34.36)	527 (54.67)	617 (34.55)	925 (49.65)	
**≥ 25^a ^(Late birth)**	573 (65.26)	501 (55.73)	596 (65.64)	437 (45.33)	1,169 (65.45)	938 (50.35)	
**Age at menopause, yr. (Mean ± SD)^b^**	49.15 ± 4.09	48.90 ± 4.43	48.71 ± 4.63	49.42 ± 4.04	48.93 ± 4.37	49.16 ± 4.25	0.27
**Menopausal status (%)^b^**							0.02
**Premenopausal**	416 (47.38)	437 (48.56)	434 (47.48)	529 (54.71)	850 (47.43)	966 (51.74)	
**Postmenopausal**	444 (50.57)	448 (49.78)	463 (50.66)	428 (44.26)	907 (50.61)	876 (46.92)	
**Estrogen receptor (ER) (%)^c^**							
Positive	369 (42.03)		434 (47.48)		803 (44.81)		
**Negative**	321 (36.56)		322 (35.23)		643 (35.88)		
**Progesterone receptor (PR) (%)^c^**							
**Positive**	396 (45.10)		414 (45.30)		810 (45.20)		
**Negative**	294 (33.49)		340 (37.20)		634 (35.38)		

^a ^10 women (0.27%) without live birth were grouped as "later birth". ^b ^Menopause information was available: 907 (51.62%) postmenopausal breast cancer cases and 876 (47.56%) postmenopausal controls. ^c ^1,446 (80.69%) ER status and 1,444 (80.58%) PR status was available in cases. ^d ^*P-*value calculated by *t' *test for the unequal variances between groups.

The results of the selected 15 SNPs and the breast cancer risk in testing set samples were presented in Table 1. The call rates of the 15 SNPs were all above 95% and the MAF in the controls were all above 0.05. Five SNPs at 2q35, 3p24, 6q22, 6q25 and 10q26 were significantly associated with breast cancer risk (2q35: rs13387042, *P *= 0.039; 3p21.4: rs2307032, *P *= 0.017; 6q22.33: rs2180341, *P *= 0.040; 6q25.1: rs2046210, *P *= 1.26 × 10^-5^; 10q26.13: rs2981582, *P *= 0.037). Therefore, these five SNPs were included in the further validation analyses.

The call rates of the five SNPs in the validation stage were all above 95% (Table 3). Consistent associations were observed for the five SNPs, with significant or borderline significant *P-*values. Overall, after adjustment for age, age at menarche, menopausal status and age at first live birth, the five SNPs showed significant associations with breast cancer susceptibility (dominant genetic model: 2q35, rs13387042: OR = 1.26, 95% CI = 1.07 to 1.49; 3q24.1, rs2307032: OR = 1.24, 95% CI = 1.07 to 1.44; 6q22.33, rs2180341: OR = 1.22, 95% CI = 1.06 to 1.40; 6q25.1, rs2046210: OR = 1.51, 95% CI = 1.31 to 1.75; 10q26.13, rs2981582: OR = 1.31, 95% CI = 1.14 to 1.50).

**Table 3:** Association of SNPs with breast cancer risk in both testing and validation sets

Genotype	**Testing Set, TS**. (*n *= 1,779)		**Validation Set, VS**. (*n *= 1,881)		Adjusted OR (95% CI)^a^	*P * ^a^	Adjusted OR (95% CI)^b^	*P * ^b^	Adjusted OR (95% CI)^c^	*P * ^c^
							
	Case (%) (*n *= 878)	Control (%) (*n *= 900)	Cases (%) (*n *= 914)	Controls (%) (*n *= 967)						
**2q35: rs13387042**										
**GG**	627 (72.74)	695 (77.91)	712 (78.33)	773 (80.60)	1.00 (ref.)		1.00 (ref.)		1.00 (ref.)	
**GA**	223 (25.87)	188 (21.08)	181 (19.91)	178 (18.56)	**1.29 (1.03 to 1.63)**	0.030	1.21 (0.93 to 1.56)	0.155	**1.23 (1.04 to 1.46)**	0.018
**AA**	12 (1.39)	9 (1.01)	16 (1.76)	8 (0.83)	1.46 (0.60 to 3.54)	0.407	**2.56 (1.03 to 6.38)**	0.044	**1.95 (1.04 to 3.66)**	0.037
**GA/AA**	235 (27.26)	197 (22.09)	197 (21.67)	186 (19.40)	**1.30 (1.04 to 1.63)**	0.023	1.27 (0.99 to 1.63)	0.064	**1.26 (1.07 to 1.49)**	0.006
**A allelic trend (Additive model)**					**1.30 (1.05 to 1.62)**	0.017	1.21 (0.97 to 1.51)	0.085	**1.25 (1.07 to 1.46)**	0.004
**3q24.1: rs2307032**										
**CC**	265 (30.78)	327 (37.16)	288 (31.58)	326 (34.42)	1.00 (ref.)		1.00 (ref.)		1.00 (ref.)	
**CT**	436 (50.64)	398 (45.23)	429 (47.04)	464 (49.00)	**1.31 (1.05 to 1.63)**	0.016	1.09 (0.87 to 1.37)	0.459	**1.20 (1.02 to 1.40)**	0.027
**TT**	160 (18.58)	155 (17.61)	195 (21.38)	157 (16.58)	1.27 (0.95 to 1.69)	0.105	**1.43 (1.06 to 1.91)**	0.018	**1.36 (1.11 to 1.66)**	0.003
**CT/TT**	596 (69.22)	553 (62.84)	624 (68.42)	621 (65.58)	**1.30 (1.06 to 1.60)**	0.014	1.18 (0.95 to 1.46)	0.138	**1.24 (1.07 to 1.44)**	0.005
**T allelic trend (Additive model)**					1.11 (0.97 to 1.27)	0.122	**1.17 (1.02 to 1.34)**	0.025	**1.14 (1.04 to 1.26)**	0.006
**6q22.33: rs2180341**										
**AA**	423 (49.76)	480 (54.55)	479 (52.64)	541 (56.53)	1.00 (ref.)		1.00 (ref.)		1.00 (ref.)	
**AG**	373 (43.88)	334 (37.95)	380 (41.76)	350 (36.57)	**1.29 (1.05 to 1.59)**	0.016	**1.26 (1.02 to 1.56)**	0.033	**1.28 (1.10 to 1.48)**	0.001
**GG**	54 (6.35)	66 (7.50)	51 (5.60)	66 (6.90)	0.96 (0.64 to 1.43)	0.847	0.87 (0.57 to 1.32)	0.505	0.93 (0.70 to 1.24)	0.614
**AG/GG**	427 (50.24)	400 (45.45)	431 (47.36)	416 (43.47)	**1.24 (1.01 to 1.51)**	0.036	1.20 (0.97 to 1.47)	0.086	**1.22 (1.06 to 1.40)**	0.006
**G allelic trend (Additive model)**					1.10 (0.94 to 1.29)	0.222	1.06 (0.90 to 1.24)	0.496	1.08 (0.97 to 1.20)	0.174
**6q25.1: rs2046210**										
**GG**	290 (33.68)	387 (43.48)	292 (32.19)	380 (39.58)	1.00 (ref.)		1.00 (ref.)		1.00 (ref.)	
**GA**	413 (47.97)	393 (44.16)	460 (50.72)	443 (46.15)	**1.46 (1.17 to 1.81)**	6.31 × 10^to 4^	**1.38 (1.10 to 1.73)**	4.81 × 10^-3^	**1.43 (1.23 to 1.67)**	5.12 × 10^-6^
**AA**	158 (18.35)	110 (12.36)	155 (17.09)	137 (14.27)	**2.05 (1.52 to 2.76)**	2.95 × 10^-6^	**1.54 (1.13 to 2.09)**	5.76 × 10^-3^	**1.79 (1.45 to 2.22)**	7.13 × 10^-8^
**GA/AA**	571 (66.32)	503 (56.52)	615 (67.81)	580 (60.42)	**1.59 (1.29 to 1.94)**	8.52 × 10^-6^	**1.42 (1.15 to 1.76)**	1.27 × 10^-3^	**1.51 (1.31 to 1.75)**	2.40 × 10^-8^
**A allelic trend (Additive model)**					**1.41 (1.22 to 1.62)**	1.54E-06	**1.24 (1.08 to 1.43)**	2.27E-03	**1.33 (1.20 to 1.46)**	1.92E-08
**10q26.13: rs2981582**										
**CC**	362 (41.51)	420 (46.88)	370 (41.29)	464 (48.95)	1.00 (ref.)		1.00 (ref.)		1.00 (ref.)	
**CT**	400 (45.87)	388 (43.30)	420 (46.88)	408 (43.04)	**1.25 (1.01 to 1.53)**	3.85 × 10^-2^	1.20 (0.97 to 1.49)	9.84 × 10^-2^	**1.24 (1.07 to 1.44)**	4.40 × 10^-3^
**TT**	110 (12.61)	88 (9.82)	106 (11.83)	76 (8.02)	**1.49 (1.07 to 2.08)**	1.76 × 10^-2^	**1.82 (1.27 to 2.62)**	1.19 × 10^-3^	**1.65 (1.29 to 2.10)**	5.75 × 10^-5^
**CT/TT**	510 (58.49)	476 (53.13)	526 (58.71)	484 (51.05)	**1.29 (1.06 to 1.57)**	1.15 × 10^-2^	**1.29 (1.05 to 1.59)**	9.84 × 10^-2^	**1.31 (1.14 to 1.50)**	1.96 × 10^-4^
**T allelic trend (Additive model)**					**1.23 (1.07 to 1.42)**	0.005	**1.30 (1.12-1.51)**	0.0005	**1.27 (1.15 to 1.41)**	4.89E-06

Results by logistic regression ^a ^in Testing Set, ^b ^in Validation Set, ^c ^in both Testing and Validation Set; ^a, b, c ^Adjust for age, age at menarche, menopausal status and age at first live birth.

**Figure 1:**
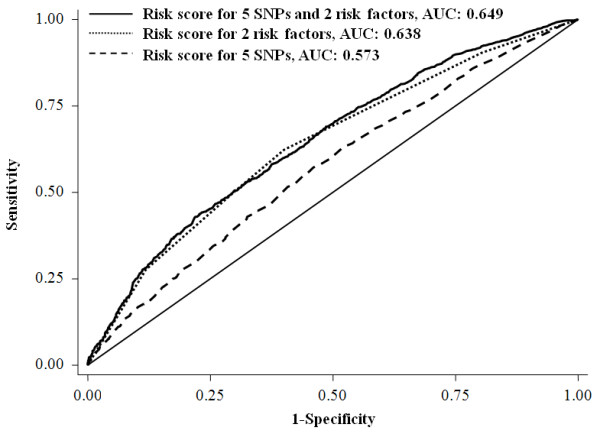
**The area under curves (AUCs) for breast cancer risk-predicting models calculated by risk score method**.

**Figure 2:**
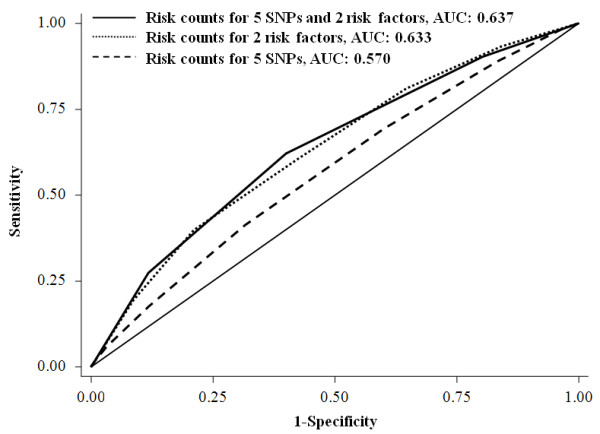
**The area under curves (AUCs) for breast cancer risk-predicting models calculated by risk counting method**.

The cumulative effects of the five SNPs and the two risk factors (age at menarche and age at first live birth) on breast cancer risk were examined by two methods (Table 4). One method was based on the counting of risk alleles/factors. Women carrying six or more risk alleles of the five SNPs (5.75% of case patients and 3.23% of control subjects) had a nearly three-fold increased risk for developing breast cancer compared with those carrying less than one of the risk alleles (11.08% of case subjects and 16.70% of control subjects). When taking age at menarche and age at first live birth into consideration, the top group (having more than seven risk alleles/factors) had a 5.61-fold increased risk compared to the reference group (adjusted OR = 5.61, 95% CI = 4.16 to 7.56). Another method was based on the risk score calculated with a linear combination of the SNP alleles or risk factors weighted by the individual odds ratio and then classified into four groups by the quartiles. Subjects with the upper quartile risk score were associated with a 91% increased breast cancer risk compared to those having the low quartile score (adjusted OR = 1.91, 95% CI = 1.56 to 2.35, *P *for trend: 5.60 × 10^-10^). Similarly, a 4.73-fold increased risk was illustrated when taking age at menarche and age at first live birth into consideration (adjusted OR = 4.73, 95% CI = 3.80 to 5.88, *P *for trend: 2.27 × 10^-47^). We then assessed the performance of the two risk prediction methods in discriminating cases and controls by ROC curves analyses. The AUC for the risk score analysis (0.649, 95% CI: 0.631 to 0.667; sensitivity = 62.60%, specificity = 57.05%, Figure 1) was significantly higher than that by the risk factors counting method (AUC: 0.637, 95% CI: 0.619 to 0.655; sensitivity = 62.16%, specificity = 60.03%, Figure 2) (*P *< 0.0001).

**Table 4:** Cumulative effects of associated SNPs and clinical risk factors on the risk of breast cancer in all samples

Risk models	TS + VS sets		Adjusted OR^c^ (95% CI)	*P * ^c^	*P *for trend^c^
				
	Cases (%)	Controls (%)			
**Counts of risk alleles^a^**					
**0 to 1**	187 (11.08)	295 (16.70)	1.00 (ref.)		
**2**	327 (19.37)	408 (23.10)	1.25 (0.98 to 1.60)	7.04E-02	
**3**	472 (27.96)	503 (28.48)	**1.48 (1.18 to 1.87)**	8.04E-04	
**4**	404 (23.93)	351 (19.88)	**1.85 (1.45 to 2.35)**	6.03E-07	
**5**	201 (11.91)	152 (8.61)	**2.09 (1.56 to 2.79)**	6.76E-07	
**≥ 6**	97 (5.75)	57 (3.23)	**2.67 (1.81 to 3.93)**	6.40E-07	2.27E-12
**Counts of risk factors^b^**					
**0 to 2**	109 (6.61)	277 (15.79)	1.00 (ref.)		
**3**	203 (12.31)	344 (19.61)	**1.54 (1.16 to 2.04)**	3.08E-03	
**4**	335 (20.32)	386 (22.01)	**2.28 (1.75 to 2.99)**	1.54E-09	
**5**	346 (20.98)	377 (21.49)	**2.47 (1.89 to 3.23)**	4.11E-11	
**6**	338 (20.50)	219 (12.49)	**4.19 (3.15 to 5.56)**	4.07E-23	
**≥ 7**	318 (19.28)	151 (8.61)	**5.61 (4.16 to 7.56)**	1.03E-29	3.76E-40
**Genetic risk score^a^**					
**0 (< Q25)**	349 (20.68)	487 (27.58)	1.00 (ref.)		
**1(Q25 to Q50)**	406 (24.05)	499 (28.26)	1.21 (0.99 to 1.48)	6.96E-02	
**2 (Q50 to Q75)**	436 (25.83)	405 (22.93)	**1.57 (1.28 to 1.93)**	1.74E-05	
**3 (≥ Q75)**	497 (29.44)	375 (21.23)	**1.91 (1.56 to 2.35)**	5.60E-10	2.02E-11
**Genetic & clinical risk score^b^**					
**0 (< Q25)**	167 (10.13)	437 (24.91)	1.00 (ref.)		
**1 (Q25 to Q50)**	332 (20.13)	442 (25.20)	**2.01 (1.60 to 2.53)**	2.68E-09	
**2 (Q50 to Q75)**	406 (24.62)	438 (24.97)	**2.55 (2.03 to 3.20)**	6.66E-16	
**3 (≥ Q75)**	744 (45.12)	437 (24.91)	**4.73 (3.80 to 5.88)**	2.38E-44	2.27E-47

^a ^Based on the five SNPs; ^b ^Based on five SNPs and two risk factors; ^c ^Adjust for age, age at menarche, menopausal status and age at first live birth where was appropriate.

**Figure 3:**
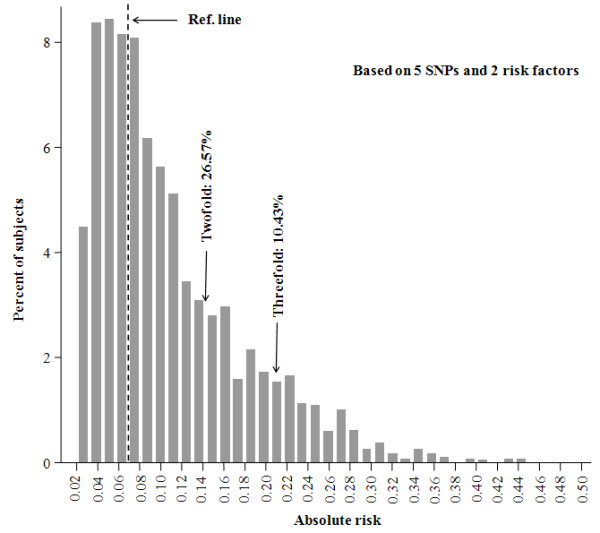
**Distribution of estimated absolute risk of breast cancer by modified Gail model in all samples**.

**Figure 4:**
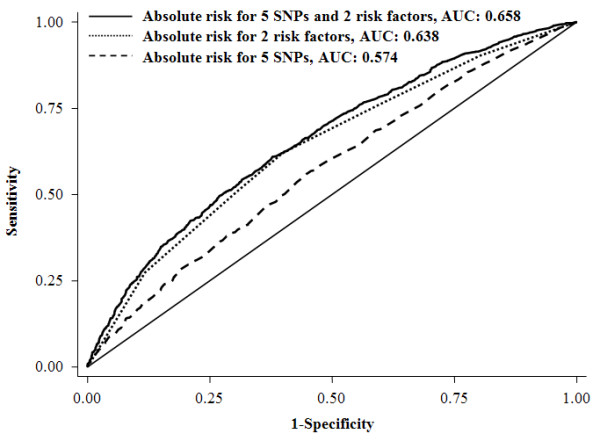
**The area under curves (AUC) for absolute risk of breast cancer**.

Absolute risk was also calculated to evaluate the combined effects of the five SNPs and the two risk factors by a modified Gail model and a 65-year absolute risk for breast cancer among women aged 20 to 85 years was estimated for each subject. From Table 5, a clear trend was observed that more subjects were grouped as high risk along with the increased numbers of risk alleles/factors. However, the variation of absolute risk distribution increased with increasing numbers of factors used in the risk-predicting model. Compared to a uniform 65-year cumulative risk 0.07 as carrying four risk factors (chosen by the largest proportion in controls: 22.01%, Table 5) for breast cancer in the population, a wide spectrum of absolute risk estimates was found using these five markers and the two clinical risk factors (Figure 3). At a cutoff of 0.14 (two-fold of the population median risk) or 0.21 (three-fold of the population median risk), 26.57% or 10.43% of women were grouped as high risk, respectively. We also used the ROC curve analysis to evaluate the performance of absolute risk to classify the cases and controls. As shown in Figure 4, we obtained an AUC of 0.658 (95% CI: 0.640 to 0.676) (sensitivity = 61.98%, specificity = 60.26%) for five SNPs plus two risk factors. Based on the cross-validation, similar results for AUCs were obtained (0.572 (five SNPs only), 0.644 (two risk factors only) and 0.660 (five SNPs plus two risk factors)), which suggests a relative reliability of the models.

**Table 5:** Absolute risk estimated in all samples

Groups	Case (%)	Controls (%)	Relative risk		Absolute risk	
			
			Median (SD)	Min to Max	Median (SD)	Min to Max
**Counts of risk alleles^a^**						
**0 to 1**	187 (11.08)	295 (16.70)	0.51 (0.05)	0.45 to 0.59	0.03 (0.003)	0.03 to 0.03
**2**	327 (19.37)	408 (23.10)	0.65 (0.06)	0.53 to 0.77	0.04 (0.003)	0.03 to 0.04
**3**	472 (27.96)	503 (28.48)	0.81 (0.08)	0.61 to 0.97	0.05 (0.005)	0.04 to 0.06
**4**	404 (23.93)	351 (19.88)	0.96 (0.11)	0.69 to 1.23	0.06 (0.006)	0.04 to 0.07
**5**	201 (11.91)	152 (8.61)	1.16 (0.12)	0.87 to 1.53	0.07 (0.007)	0.05 to 0.09
**≥ 6**	97 (5.75)	57 (3.23)	1.45 (0.21)	1.10 to 2.36	0.08 (0.012)	0.06 to 0.14
**Counts of risk factors^b^**						
**0 to 2**	109 (6.61)	277 (15.79)	0.61 (0.20)	0.35 to 1.14	0.04 (0.01)	0.02 to 0.07
3	203 (12.31)	344 (19.61)	0.91 (0.33)	0.47 to 2.00	0.05 (0.02)	0.03 to 0.12
**4**	335 (20.32)	386 (22.01)^c^	1.22 (0.47)	0.53 to 2.62	0.07 (0.03)	0.03 to 0.15
**5**	346 (20.98)	377 (21.49)	1.78 (0.65)	0.67 to 3.43	0.10 (0.04)	0.04 to 0.20
**6**	338 (20.50)	219 (12.49)	2.44 (0.82)	0.88 to 4.33	0.14 (0.05)	0.05 to 0.25
**≥ 7**	318 (19.28)	151 (8.61)	3.92 (1.24)	1.11 to 7.78	0.23 (0.07)	0.06 to 0.45

^a ^Based on the five SNPs; ^b ^Based on five SNPs and two risk factors; ^c ^Chose as ref. group.

The stratified analyses by ER or PR status of the five SNPs were summarized in Additional file 2. However, no significant heterogeneity was observed for the effect of each SNP by different ER or PR subgroups. Further stratified analysis was conducted on the cumulative effects of the five SNPs (coded 0 to 2 risk alleles as 0 and more than 3 risk alleles as 1) and found no heterogeneity between subgroups (Additional file 3).

## Discussion

In our study involving 1,792 breast cancer cases and 1,867 cancer-free controls, 5 of the 15 variants, identified in previous GWAS studies [8-14], were consistently associated with breast cancer risk in this Chinese population. Risk assessment models and absolute risk calculations combining the five SNPs and two clinical risk factors indicated the small effects of these markers in discriminating cases and controls. Overall, the results provide further evidence and utility for GWAS identified SNPs in relation to breast cancer risk assessment in Chinese women.

We summarized associations of the 15 SNPs of breast cancer identified by previous GWAS studies and following replication studies (Additional file 4). SNP rs13387042 at 2q35 was identified as a breast cancer susceptibility SNP in two GWAS conducted among Europeans [12,13]. Significant associations were also observed in most of the later studies on Europeans and African American women [30-36] except for one reported by Stevens KN *et al. *[37]. However, the results were conflicting in Asian populations [12,17,38,39]. For 3p24, Ahmed *et al. *reported marker SNPs rs4973768 and rs1357245 in a four-stage GWAS study, and then located the strongest marker rs2307032 in this region [8]. Following replication studies for 3p24 region also presented consistent results among European [34-37] and Asian [38,40], including our study. SNP rs2180341 at 6q21.33 was originally found in the Ashkenazi Jewish population [10] and was well replicated in Europeans [41]. In the current study, we found consistent results among Chinese; however, no significant association was observed in other studies involving Asian populations [17,31,36,38]. SNP rs2046210, located at upstream of the *ESR1 *gene on chromosome 6q25.1, was the only one reported by Zheng *et al. *(2009) in a GWAS conducted among Chinese women [14] and consistently replicated in Asian populations (Chinese and Japanese women, including partly overlapped samples from our group) [17,42-44] and women of European-ancestry [14,36,37,42], but not in African American women [31,44]. SNP rs2981582 (10q26.13) was reported by Easton *et al. *in the first large-scale breast cancer GWAS [10], which was replicated in Europeans and Asians [17,32-36,38,40,45-47], and was also reported previously with partly overlapped study samples by our group [25], but not in Africans [31,46]. In the current study, we enlarged our study subjects and obtained similar results.

For the other SNPs, Han *et al. *successfully replicated SNPs rs4973768 (3p24.1), rs889312 (5p11.2) and rs3803662 (16q12.1) in Korean women with breast cancer [40]. However, SNPs rs4973768 (3q24.1), rs10941679 (5p12), rs889312 (5p11.2), rs13281615 (8q24.21), rs3817198 (11p15.5), rs12443621 (16q12.1) and rs6504950 (17q23.2) were not reported to be associated with breast cancer in Chinese women [17,24,38,39], which was similar to our results. Potential explanations for the failure of replication of these SNPs in Chinese could be the genetic heterogeneity (both allelic and locus heterogeneity). Allelic heterogeneity is the phenomenon in which different mutations at the same locus (or gene) cause the same disorder. While locus heterogeneity implies that mutation in different genes may explain one variant phenotype. Further large scale resequencing or fine mapping studies on these regions may help find breast cancer causal variants.

Traditional approaches to assessing patients' disease risk are primarily achieved through non-genetic risk factors with apparent limitations, and it is expected that a better prediction can be reached if we can incorporate genetic determinants. Recently, several studies on these efforts were published [16-22]. Zheng *et al. *conducted a validation study with 3,039 breast cancer cases and 3,082 controls for 12 GWAS identified SNPs (nine regions) in Asian women [17], and built a risk assessment model with eight SNPs and five clinical risk factors. However, only five of the eight SNPs were significantly associated with breast cancer susceptibility in the study. In our current study, two more regions were incorporated (3q24.1, 17q23.2) and we found five susceptibility SNPs with a two-stage validations, although the performance of the risk assessment model was still limited.

Overall, risk model prediction is not a diagnostic tool but provides an estimate of likelihood of developing disease in the future. A well-evaluated risk model, taking genetic and clinical risk factors together, can be used as a screening tool for high risk individuals among the general population. Women at high risk for breast cancer can be focused on by choosing an optimal cutoff (for example, two-fold of the population median risk), and these women should perform regular breast cancer screening [48,49]. Results from this study suggest that GWAS identified SNPs can be used to improve the prediction model. However, there are a number of limitations for the current study. First, several newly reported breast cancer risk-associated SNPs were not included in the current analysis [50]. Second, more breast cancer associated risk factors should be evaluated, such as the body mass index (BMI) and family history of breast cancer [14]. However, the effects on breast cancer risk by BMI could not be well-evaluated in our study with a retrospective study design. Our moderate study sample size limited our power to evaluate the parameters of breast cancer family history (only 101 cases (7.39%) and 3 controls (0.29%) with a positive breast cancer family history). Third, the two-stage study design, although helping to avoid false positive findings, may cause the omission of low but true associations, because our overall study sample size is moderate.

## Conclusions

Overall, five GWAS identified variants were also consistently validated in this Chinese population. Risk assessment models that incorporate both a genetic risk score based on these SNPs and the established risk factors for breast cancer may be useful for identifying high-risk women for targeted cancer prevention. More genetic risk variants and other risk factors should be well evaluated and incorporated into the risk-predicting models to improve the ability of personalized risk assessment.

## Abbreviations

AR: absolute risk; AUC: area under the curve; CHB: Chinese Han Beijing; CI: confidence intervals; ER: estrogen receptor; GWAS: genome-wide association studies; LD: linkage disequilibrium; MAF: minor allele frequency; ORs: odds ratios; PR: progesterone receptor; ROC curves: receiver-operator characteristic curves; SNPs: single nucleotide polymorphisms

## Competing interests

The authors declare that they have no competing interests.

## Authors' contributions

HS directed the study, obtained financial support, and was responsible for study design, interpretation of results and manuscript writing. JD performed data management, statistical analyses and drafted the initial manuscript. ZH performed overall project management and manuscript writing. YJ, HS, JD and HM were responsible for samples processing and managed the genotyping data. All authors read and approved the final manuscript.

## Supplementary Material

Additional file 1 **Supplementary Figure **1. Analysis workflow. Click here for file

Additional file 2 **Supplementary Figure **2. Association of five SNPs with breast cancer risk in all the study samples, stratified by estrogen receptor (ER), Progesterone receptor (PR) status. Click here for file

Additional file 3 **Supplementary Figure **3. Stratification analysis of cumulative effects about the five SNPs with breast cancer risk in all samples. Click here for file

Additional file 4 **Supplementary Table **1. Associations of the 15 SNPs of breast cancer identified by previous GWAS studies and following replicated association studies. Click here for file
