# Supplementary figures and images for: Breast cancer risk assessment with five independent genetic variants and two risk factors in Chinese women
Source: Breast Cancer Res. 2012 Jan 23;14(1):R17. doi: 10.1186/bcr3101 (PMC3496134; doi:10.1186/bcr3101)

**Supplementary Figure 1.** Analysis workflow.


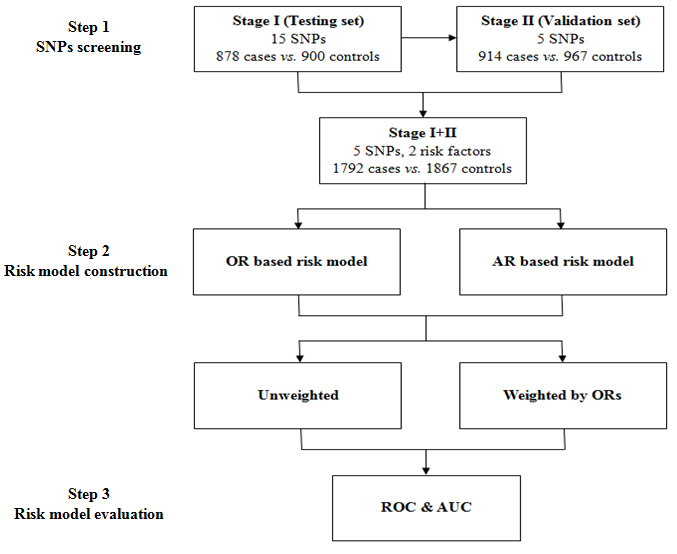

Supplement: Additional file 1 — Supplementary Figure 1. Analysis workflow. [file bcr3101-S1.DOC]
