# Supplementary material for: Breast cancer risk assessment with five independent genetic variants and two risk factors in Chinese women
Source: Breast Cancer Res. 2012 Jan 23;14(1):R17. doi: 10.1186/bcr3101 (PMC3496134; doi:10.1186/bcr3101)
Supplement: Additional file 2 — Supplementary Figure 2. Association of five SNPs with breast cancer risk in all the study samples, stratified by estrogen receptor (ER), Progesterone receptor (PR) status. [file bcr3101-S2.DOC]

**Supplementary Figure 2.** Association of 5 SNPs with breast cancer risk in all the study samples, stratified by Estrogen receptor (ER), Progesterone receptor (PR) status. Adjust for age, age at menarche and age at first live birth child. *P* values in the plot for heterogeneity between groups by chi-square based *Q* tests.

**1.24 (1.09, 1.41)**

**1.36 (1.10, 1.67)**

1.11 (0.97, 1.27)

**1.30 (1.14, 1.47)**

**1.31 (1.15, 1.51)**

**1.25 (1.03, 1.51)**

1.03 (0.89, 1.20)

**1.26 (1.09, 1.45)**

**OR (95% CI)**

**1.34 (1.17, 1.53)**

1.04 (0.92, 1.17)

1

1

1.67

0.056

0.689

0.572

0.337

0.241

**Stratified by PR Status**

***P* value**

0.538

0.463

0.936

0.559

0.055

***P* value**

Negative

Positive

**6q25.1: rs2046210**

Negative

**10q26.13: rs2981582**

**5 SNPs Stratified by ER Status**

**3q24.1: rs2307032**

Positive

Negative

**6q22.33: rs2180341**

Negative

Positive

Positive

Negative

Positive

**2q35: rs13387042**

**1.18 (1.02, 1.36)**

1.07 (0.95, 1.21)

**1.19 (1.05, 1.36)**

**OR (95% CI)**

1.05 (0.91, 1.21)

1.10 (0.95, 1.27)

**1.35 (1.18, 1.55)**

1.21 (1.00, 1.48)

**1.28 (1.13, 1.45)**

**1.39 (1.14, 1.71)**

**1.43 (1.25, 1.63)**

1

1

1.71
