# Supplementary material for: Breast cancer risk assessment with five independent genetic variants and two risk factors in Chinese women
Source: Breast Cancer Res. 2012 Jan 23;14(1):R17. doi: 10.1186/bcr3101 (PMC3496134; doi:10.1186/bcr3101)
Supplement: Additional file 3 — Supplementary Figure 3. Stratification analysis of cumulative effects about the five SNPs with breast cancer risk in all samples. [file bcr3101-S3.DOC]

**Supplementary Figure 3.** Stratification analysis of cumulative effects about the 5 SNPs with breast cancer risk in all samples. Adjust for age, age at menarche and age at first live birth where was appropriate. *P* values in the plot for heterogeneity between groups by chi-square based *Q* tests.

**Estrogen receptor (ER)**

Postmenopausal

**Age at menarche, yr.**

15-17

Negative

Positive

**Menopausal status**

**Progesterone receptor (PR)**

Positive

Premenopausal

≥25

**Stratified Variables**

≥50

Negative

<25

<50

<15

**Age at first live birth, yr.**

**Age group, yr.**

**1.70 (1.38, 2.09)**

**1.61 (1.28, 2.02)**

**1.65 (1.34, 2.02)**

**1.52 (1.25, 1.83)**

**1.49 (1.24, 1.80)**

**1.42 (1.15, 1.75)**

**1.52 (1.25, 1.84)**

**OR (95% CI)**

**1.61 (1.31, 1.98)**

**1.68 (1.36, 2.07)**

**1.56 (1.25, 1.95)**

**1.50 (1.22, 1.85)**

**1.53 (1.17, 2.00)**

1

1

2.09

0.538

0.863

0.232

0.636

0.956

***P* value**

0.566

≥17

**1.50 (1.16, 1.96)**
