# Supplementary material for: Breast cancer risk assessment with five independent genetic variants and two risk factors in Chinese women
Source: Breast Cancer Res. 2012 Jan 23;14(1):R17. doi: 10.1186/bcr3101 (PMC3496134; doi:10.1186/bcr3101)
Supplement: Additional file 4 — Supplementary Table 1. Associations of the 15 SNPs of breast cancer identified by previous GWAS studies and following replicated association studies. [file bcr3101-S4.DOC]

**Supplementary Table 1**. Associations of the 15 SNPs of breast cancer identified by previous GWAS studies and following replicated association studies.

| **First Author** | **Year** | **PMID** | **Population** | **Type** | **Cases/Controls** | **OR (%95CI) ^a^** | **Ref.** |
| --- | --- | --- | --- | --- | --- | --- | --- |
| **2q35: rs13387042, G>A, MAF ^b^: CHB 0.11, CEU 0.56, YRI 0.76.** | | | | | | |  |
| **Stacey SN** | **2007** | **17529974** | **European** | **GWAS** | **4533/17513** | **1.20(1.14-1.26)** | **[12]** |
| **Thomas G** | **2009** | **19330030** | **European** | **GWAS** | **5285/5433** | **1.35(1.27-1.43)** | **[13]** |
| Milne RL | 2009 | 19567422 | European | Replication | 31510/35969 | **1.12(1.09-1.15)** | [30] |
| Zheng W | 2009 | 19789366 | African | Replication | 810/1784 | **1.20(1.03-1.39)** | [31] |
| Zheng W | 2010 | 20484103 | Chinese | Replication | 3039/3082 | 1.03(0.92-1.16) | [17] |
| Travis RC | 2010 | 20605201 | European | Replication | 7610/10196 | **1.16(1.11-1.21)** | [32] |
| Reeves GK | 2010 | 20664043 | European | Replication | 10306/10393 | **1.16(1.11-1.21)** | [33] |
| Long J | 2010 | 20699374 | Chinese | Replication | 6498/3999 | 1.03(0.92-1.16) | [38] |
| Milne RL | 2010 | 21194473 | Mixed | Replication | 32917/25996 | **1.14(1.11-1.17)** | [34] |
| Jiang Y | 2011 | 21197568 | Chinese | Replication | 492/510 | 1.26(0.95-1.67) | [39] |
| Broeks A | 2011 | 21596841 | European | Replication | 30040/53692 | **1.14(1.12-1.17)** | [35] |
| Campa D | 2011 | 21791674 | European | Replication | 8576/11892 | **1.18(1.03-1.32)** | [36] |
| Stevens KN | 2011 | 21844186 | European | Replication | 2977/4976 | 0.96(0.90-1.03) | [37] |
| **3p24.1: rs4973768, C>T, MAF ^b^: CHB 0.17, CEU 0.44, YRI 0.30.** | | | | | | |  |
| **Ahmed S** | **2009** | **19330027** | **European** | **GWAS** | **30256/34063** | **1.11(1.08-1.13)** | **[8]** |
| Long J | 2010 | 20699374 | Chinese | Replication | 6498/3999 | **1.12(1.04-1.21)** | [38] |
| Milne RL | 2010 | 21194473 | Mixed | Replication | 30366/22929 | **1.11(1.09-1.14)** | [34] |
| Han W | 2011 | 21415360 | Korean | Replication | 3321/3500 | **1.20(1.08-1.33)** | [40] |
| Broeks A | 2011 | 21596841 | European | Replication | 30040/53692 | **1.11(1.09-1.14)** | [35] |
| Campa D | 2011 | 21791674 | European | Replication | 8576/11892 | **1.08(1.04-1.13)** | [36] |
| Stevens KN | 2011 | 21844186 | European | Replication | 2960/4974 | 1.04(0.97-1.12) | [37] |
| **3p24.1: rs2307032,** **C>T, MAF ^b^: CHB 0.41, CEU 0.64, YRI 1.00.** | | | | | | |  |
| **Ahmed S** | **2009** | **19330027** | **European** | **GWAS** | **30256/34063** | **0.88(0.80-0.96)** | **[8]** |
| **5p11.2: rs16886165, T>G, MAF ^b^: CHB 0.31, CEU 0.16, YRI 0.32.** | | | | | | |  |
| **Thomas G** | **2009** | **19330030** | **European** | **GWAS** | **5283/5440** | **1.65(1.30-2.10)** | **[13]** |
| **5q11.2: rs889312,** **C>A, MAF ^b^: CHB 0.50, CEU 0.69, YRI 0.67.** | | | | | | |  |
| **Easton DF** | **2007** | **17529967** | **European** | **GWAS** | **21668/2093** | **1.13(1.10-1.16)** | **[9]** |
| Garcia-Closas M | 2008 | 18437204 | European | Replication | 20455/25331 | **1.12(1.09-1.15)** | [45] |
| Garcia-Closas M | 2008 | 18437204 | Chinese | Replication | 892/750 | 1.12(0.97-1.28) | [45] |
| Rebbeck TR | 2009 | 19028704 | European | Replication | 528/697 | 1.10(0.90-1.35) | [46] |
| Rebbeck TR | 2009 | 19028704 | African | Replication | 157/427 | 1.33(0.98-1.81) | [46] |
| Zheng W | 2009 | 19789366 | African | Replication | 810/1784 | 0.98(0.85-1.12) | [31] |
| Zheng W | 2010 | 20484103 | Chinese | Replication | 3039/3082 | 1.07(0.99-1.15) | [17] |
| Travis RC | 2010 | 20605201 | European | Replication | 7610/10196 | **1.13(1.08-1.19)** | [32] |
| Reeves GK | 2010 | 20664043 | European | Replication | 10306/10393 | **1.13(1.06-1.18)** | [33] |
| Long J | 2010 | 20699374 | Chinese | Replication | 6498/3999 | 1.07(0.99-1.15) | [38] |
| Milne RL | 2010 | 21194473 | Mixed | Replication | 26227/23307 | **1.11(1.08-1.15)** | [34] |
| Han W | 2011 | 21415360 | Korean | Replication | 3321/3500 | **1.22(1.06-1.41)** | [40] |
| Broeks A | 2011 | 21596841 | European | Replication | 30040/53692 | **1.11(1.08-1.14)** | [35] |
| Jiang Y | 2011 | 21197568 | Chinese | Replication | 492/510 | 0.99(0.83-1.18) | [39] |
| Campa D | 2011 | 21791674 | European | Replication | 8576/11892 | **1.10(1.05-1.15)** | [36] |
| Stevens KN | 2011 | 21844186 | European | Replication | 2844/2757 | 1.07(0.98-1.17) | [37] |
| **5p12: rs4415084,** **A>G, MAF ^b^: CHB 0.46, CEU 0.62, YRI 0.36.** | | | | | | |  |
| **Stacey SN** | **2008** | **18438407** | **European** | **GWAS** | **5028/32090** | **1.16(1.10-1.21)** | **[12]** |
| **Thomas G** | **2009** | **19330030** | **European** | **GWAS** | **10293/9367** | **1.20(1.11-1.31)** | **[13]** |
| Campa D | 2011 | 21791674 | European | Replication | 8576/11892 | **1.08(1.03-1.12)** | [36] |
| **5p12: rs10941679,** **G>A, MAF ^b^: CHB 0.43, CEU 0.76, YRI 0.83.** | | | | | | |  |
| **Stacey SN** | **2008** | **18438407** | **European** | **GWAS** | **5028/32090** | **1.19(1.13-1.26)** | **[12]** |
| **Thomas G** | **2009** | **19330030** | **European** | **GWAS** | **5490/4575** | **1.20(1.03-1.41)** | **[13]** |
| Zheng W | 2009 | 19789366 | African | Replication | 810/1784 | 0.98(0.83-1.15) | [31] |
| Zheng W | 2010 | 20484103 | Chinese | Replication | 3039/3082 | 1.07(0.99-1.15) | [17] |
| Milne RL | 2010 | 21194473 | Mixed | Replication | 31513/25008 | **1.12(1.09-1.15)** | [34] |
| Campa D | 2011 | 21791674 | European | Replication | 8576/11892 | **1.12(1.07-1.17)** | [36] |
| Stevens KN | 2011 | 21844186 | European | Replication | 2705/1385 | 1.04(0.94-1.16) | [37] |
| **6q22.33: rs2180341,** **A>G, MAF ^b^: CHB 0.22, CEU 0.26, YRI 0.36.** | | | | | | |  |
| **Gold B** | **2008** | **18326623** | **Ashkenazi Jews** | **GWAS** | **1442/1465** | **1.41(1.25-1.59)** | **[10]** |
| Kirchhoff T | 2009 | 19690183 | European | Replication | 3031/2616 | **1.24(1.13-1.36)** | [41] |
| Zheng W | 2009 | 19789366 | African | Replication | 810/1784 | 1.06(0.93-1.22) | [31] |
| Zheng W | 2010 | 20484103 | Chinese | Replication | 3039/3082 | 0.93(0.86-1.02) | [17] |
| Long J | 2010 | 20699374 | Chinese | Replication | 6498/3999 | 0.94(0.86-1.02) | [38] |
| Campa D | 2011 | 21791674 | European | Replication | 8576/11892 | 0.96(0.91-1.01) | [36] |
| **6q25.1: rs2046210,** **G>A, MAF ^b^: CHB 0.35, CEU 0.29, YRI 0.70.** | | | | | | |  |
| **Zheng W** | **2009** | **19219042** | **Chinese** | **GWAS** | **6472/3962** | **1.29(1.21-1.37)** | **[14]** |
| **Zheng W** | **2009** | **19219042** | **European** | **GWAS** | **1591/1466** | **1.15(1.03-1.28)** | **[14]** |
| Zheng W | 2009 | 19789366 | African | Replication | 810/1784 | 1.00(0.87-1.14) | [31] |
| Zheng W | 2010 | 20484103 | Chinese | Replication | 3039/3082 | **1.26(1.17-1.35)** | [17] |
| Stacey SN | 2010 | 20661439 | Asian | Replication | 1126/1118 | **1.24(1.10-1.40)** | [44] |
| Stacey SN | 2010 | 20661439 | European | Replication | 7899/11234 | 1.04(0.99-1.08) | [44] |
| Stacey SN | 2010 | 20661439 | African | Replication | 1151/934 | 0.98(0.86-1.11) | [44] |
| Long J | 2010 | 20699374 | Chinese | Replication | 6498/3999 | **1.28(1.21-1.36)** | [38] |
| Jiang Y | 2011 | 21197568 | Chinese | Replication | 492/510 | **1.42(1.18-1.71)** | [39] |
| Han W | 2011 | 21415360 | Korean | Replication | 3321/3500 | **1.29(1.16-1.43)** | [40] |
| Campa D | 2011 | 21791674 | European | Replication | 8576/11892 | **1.10(1.05-1.15)** | [36] |
| Stevens KN | 2011 | 21844186 | European | Replication | 2707/1385 | **1.29(1.17-1.42)** | [37] |
| Cai Q | 2011 | 21303983 | Chinese | Replication | 1983/1203 | **1.28(1.23-1.33)** | [42] |
| Cai Q | 2011 | 21303983 | European | Replication | 587/473 | **1.07(1.01-1.14)** | [42] |
| Han J | 2011 | 21528353 | Chinese | Replication | 1186/1083 | **1.32(1.20-1.46)** | [43] |
| **8q24.21: rs13281615,** **A>G, MAF ^b^: CHB 0.57, CEU 0.46, YRI 0.43.** | | | | | | |  |
| **Easton DF** | **2007** | **17529967** | **European** | **GWAS** | **21668/2093** | **1.08(1.05-1.11)** | **[9]** |
| Garcia-Closas M | 2008 | 18437204 | European | Replication | 18422/21354 | **1.12(1.09-1.15)** | [45] |
| Garcia-Closas M | 2008 | 18437204 | Chinese | Replication | 896/751 | 0.98(0.86-1.13) | [45] |
| Zheng W | 2009 | 19789366 | African | Replication | 810/1784 | 1.02(0.91-1.15) | [31] |
| Zheng W | 2010 | 20484103 | Chinese | Replication | 3039/3082 | 1.06(0.98-1.14) | [17] |
| Travis RC | 2010 | 20605201 | European | Replication | 7610/10196 | **1.08(1.04-1.13)** | [32] |
| Reeves GK | 2010 | 20664043 | European | Replication | 10306/10393 | **1.09(1.05-1.14)** | [33] |
| Long J | 2010 | 20699374 | Chinese | Replication | 6498/3999 | 1.06(0.98-1.14) | [38] |
| Milne RL | 2010 | 21194473 | Mixed | Replication | 21823/20609 | **1.12(1.09-1.15)** | [30] |
| Jiang Y | 2011 | 21197568 | Chinese | Replication | 492/510 | 1.01(0.85-1.21) | [39] |
| Broeks A | 2011 | 21596841 | European | Replication | 30040/53692 | **1.11(1.08-1.13)** | [35] |
| Campa D | 2011 | 21791674 | European | Replication | 8576/11892 | **1.09(1.05-1.13)** | [36] |
| Stevens KN | 2011 | 21844186 | European | Replication | 2841/3413 | 0.99(0.92-1.07) | [37] |
| **8q24.21: rs1562430,** **T>C, MAF ^b^: CHB 0.20, CEU 0.35, YRI 0.49.** | | | | | | |  |
| **Thomas G** | **2009** | **19330030** | **European** | **GWAS** | **5285/5440** | **1.27(1.18-1.36)** | **[13]** |
| **Turnbull C** | **2010** | **20453838** | **European** | **GWAS** | **3659/4897** | **1.17(1.10-1.25)** | **[50]** |
| **10q26.13: rs2981582,** **C>T, MAF ^b^: CHB 0.33, CEU 0.46, YRI 0.51.** | | | | | | |  |
| **Easton DF** | **2007** | **17529967** | **European** | **GWAS** | **21668/2093** | **1.26(1.23-1.30)** | **[10]** |
| Garcia-Closas M | 2008 | 18437204 | European | Replication | 20410/25314 | **1.26(1.23-1.30)** | [45] |
| Garcia-Closas M | 2008 | 18437204 | Chinese | Replication | 888/744 | **1.32(1.14-1.53)** | [45] |
| Liang J | 2008 | 18845558 | Chinese | Replication | 1049/1073 | **1.62(1.19-2.20)** | [25] |
| Rebbeck TR | 2009 | 19028704 | European | Replication | 528/697 | **1.26(1.04-1.53)** | [46] |
| Rebbeck TR | 2009 | 19028704 | African | Replication | 157/427 | 0.80(0.59-1.08) | [46] |
| Boyarskikh UA | 2009 | 19536173 | Russian | Replication | 766/665 | **1.46(1.30-1.62)** | [47] |
| Zheng W | 2009 | 19789366 | African | Replication | 810/1784 | 1.03(0.91-1.18) | [31] |
| Zheng W | 2010 | 20484103 | Chinese | Replication | 3039/3082 | **1.15(1.07-1.25)** | [17] |
| Travis RC | 2010 | 20605201 | European | Replication | 7610/10196 | **1.22(1.17-1.28)** | [32] |
| Long J | 2010 | 20699374 | Chinese | Replication | 6498/3999 | **1.14(1.07-1.22)** | [38] |
| Reeves GK | 2010 | 20664043 | European | Replication | 10306/10393 | **1.23(1.18-1.28)** | [33] |
| Milne RL | 2010 | 21194473 | Mixed | Replication | 25821/22551 | **1.22(1.19-1.26)** | [34] |
| Han W | 2011 | 21415360 | Korean | Replication | 3321/3500 | **1.40(1.18-1.68)** | [40] |
| Broeks A | 2011 | 21596841 | European | Replication | 30040/53692 | **1.22(1.19-1.25)** | [35] |
| Campa D | 2011 | 21791674 | European | Replication | 8576/11892 | **1.21(1.16-1.26)** | [36] |
| Stevens KN | 2011 | 21844186 | European | Replication | 2707/2756 | 0.95(0.88-1.03) | [37] |
| **11p15.5: rs3817198,** **T>C, MAF ^b^: CHB 0.09, CEU 0.33, YRI 0.10.** | | | | | | |  |
| **Easton DF** | **2007** | **17529967** | **European** | **GWAS** | **21668/2093** | **1.07(1.04-1.11)** | **[10]** |
| Garcia-Closas M | 2008 | 18437204 | European | Replication | 20426/25266 | **1.07(1.04-1.10)** | [45] |
| Garcia-Closas M | 2008 | 18437204 | Chinese | Replication | 886/746 | 1.02(0.84-1.24) | [45] |
| Zheng W | 2009 | 19789366 | African | Replication | 810/1784 | 0.97(0.82-1.16) | [31] |
| Zheng W | 2010 | 20484103 | Chinese | Replication | 3039/3082 | **1.12(1.00-1.24)** | [17] |
| Travis RC | 2010 | 20605201 | European | Replication | 7610/10196 | 1.03(0.98-1.07) | [32] |
| Long J | 2010 | 20699374 | Chinese | Replication | 6498/3999 | **1.11(1.01-1.21)** | [38] |
| Milne RL | 2010 | 21194473 | Mixed | Replication | 25004/21596 | **1.08(1.05-1.11)** | [34] |
| Jiang Y | 2011 | 21197568 | Chinese | Replication | 492/510 | 0.91(0.70-1.18) | [39] |
| Broeks A | 2011 | 21596841 | European | Replication | 30040/53692 | **1.06(1.03-1.09)** | [35] |
| Campa D | 2011 | 21791674 | European | Replication | 8576/11892 | 1.00(0.95-1.04) | [36] |
| Stevens KN | 2011 | 21844186 | European | Replication | 2929/4756 | 1.03(0.95-1.10) | [37] |
| **16q12.1: rs12443621,** **G>A, MAF ^b^: CHB 0.39, CEU 0.46, YRI 0.49.** | | | | | | |  |
| **Easton DF** | **2007** | **17529967** | **European** | **GWAS** | **21668/2093** | **1.11(1.08-1.14)** | **[10]** |
| Zheng W | 2009 | 19789366 | African | Replication | 810/1784 | 0.99(0.88-1.13) | [31] |
| Liang J | 2010 | 20213080 | Chinese | Replication | 1049/1073 | 1.12(0.90-1.40) | [24] |
| Zheng W | 2010 | 20484103 | Chinese | Replication | 3039/3082 | 0.99(0.92-1.06) | [17] |
| Long J | 2010 | 20699374 | Chinese | Replication | 6498/3999 | 0.99(0.92-1.06) | [38] |
| Jiang Y | 2011 | 21197568 | Chinese | Replication | 492/510 | 0.90(0.75-1.08) | [39] |
| **17q23.2: rs6504950,** **G>A, MAF ^b^: CHB 0.10, CEU 0.31, YRI 0.31.** | | | | | | |  |
| **Ahmed S** | **2009** | **19330027** | **European** | **GWAS** | **30256/34063** | **0.95(0.92-0.97)** | **[8]** |
| Long J | 2010 | 20699374 | Chinese | Replication | 6498/3999 | 1.00(0.89-1.11) | [38] |
| Milne RL | 2010 | 21194473 | Mixed | Replication | 30045/23943 | **0.95(0.92-0.97)** | [34] |
| Broeks A | 2011 | 21596841 | European | Replication | 30040/53692 | **0.94(0.92-0.97)** | [35] |
| Campa D | 2011 | 21791674 | European | Replication | 8576/11892 | **0.92(0.88-0.97)** | [36] |
| Stevens KN | 2011 | 21844186 | European | Replication | 2707/1385 | **0.97(0.87-1.07)** | [37] |

# ^a^ The results of additive model (when additive models were not available, the results of homozygousity were extracted);

# ^b^ Minor Allele Frequency(MAF) of Chinese Han population from Beijing (CHB) based on the International HapMap project (Phase II+III, rel 27).
